# Distinguishing Lytic and Temperate Infection Dynamics in the Environment

**DOI:** 10.3390/v17040513

**Published:** 2025-04-01

**Authors:** Isha Tripathi, Naomi Barber-Choi, Lauren Woodward, Natalie Falta, Natalia Shahwan, Nickie Yang, Ben Knowles

**Affiliations:** 1 Department of Ecology and Evolutionary Biology, University of California, Los Angeles, CA 90095, USA; 2 Institute for Quantitative and Computational Biosciences, University of California, Los Angeles, CA 90095, USA; 3 Department of Computational Medicine, David Geffen School of Medicine, University of California, Los Angeles, CA 90095, USA; 4 California NanoSystems Institute, University of California, Los Angeles, CA 90095, USA; 5 Institute of the Environment and Sustainability, University of California, Los Angeles, CA 90095, USA

**Keywords:** viral ecology, theoretical model, temperate, lytic

## Abstract

Viral infection and lysis drive bacterial diversity and abundances, ultimately regulating global biogeochemical cycles. Infection can follow lytic or temperate routes, with lytic dynamics suppressing bacterial population growth and temperate infection enhancing it. Given that bacterial over-proliferation is a pervasive threat to ecosystems, determining which infection dynamic dominates a given ecosystem is a central question in viral ecology. However, the fields that describe and test the rules of viral infection—theoretical ecology and environmental microbiology, respectively—remain disconnected. To address this, we simulated common empirical approaches to analyze and distinguish between the predictions of three theoretical models mechanistically representing lytic to temperate infection dynamics. By doing so, we found that the models have remarkably similar predictions despite their mechanistic differences, as shown by PCA and correlation analyses. Essentially, the models are only discernable under simulated nutrient addition, where lytic models become less stable with no increase in host densities while the temperate model remains stable and has elevated host abundances. Highlighting this difference between the models, we present a dichotomous key illustrating how researchers can determine whether lytic or temperate infection dynamics dominate their ecosystem of interest using common metrics and empirical approaches.

## 1. Introduction

Viral infection drives host diversity, densities and growth, and global biogeochemical cycles [1,2,3]. This can occur through lytic infections where the viruses infect cells and then immediately replicate and lyse them, killing off infection-sensitive lineages [4,5]. Or it can proceed through temperate infection where viruses infect and then persist within cells for extended periods [6,7], allowing cells prolonged access to genes shared between hosts by the viruses. Altogether, this means that lytic dynamics can suppress bacterial population growth and abundances while temperate infection can enhance it [8].

In lytic dynamics, infection and lysis can be thought of and modeled as synonymous [5,9,10], similar to how predation is modeled in microorganismal predator–prey systems [11,12]. In this scenario, virus–host encounters lead to infection at a given efficiency that then leads to an equal amount of lysis because lytic viruses rapidly kill any cells they infect. This means that lytic dynamics are directly driven by encounter rates that are the product of virus and host densities. In contrast to density-dependent lytic dynamics, temperate infection appears to be modulated by host physiology, where lysis is suspended post-infection so long as hosts remain healthy [8,13,14]. This effectively decouples infection and lysis in temperate dynamics.

Given that bacterial over-proliferation is a pervasive threat to ecosystems [15], knowing which infection dynamic dominates a given ecosystem and predicting what the ecosystem impacts might be are central questions in viral ecology. However, the fields that describe and that test the rules of viral infection—theoretical ecology and environmental microbiology, respectively—remain distant. This is largely driven by a lack of overlap in expertise between empiricists and modelers that can result in empiricists avoiding the mathematics of models and in theoreticians making suggestions that are not empirically tractable [16]. Further, difficulties in communication between empirical and theoretical researchers may hint at even deeper challenges, and the possibility that empirical and theoretical fields may currently be somewhat incommensurate [17,18,19,20]. Altogether, the decoupling of theory and data prohibits the improvement and refinement of models through empirical challenges, which in turn precludes the development of an empirically sound predictive understanding of viral infections and effects.

To remedy this, we examined three theoretical models mechanistically representing lytic, super-lytic, and sub-lytic/temperate infection dynamics. These models either had lysis rates that were unaffected, amplified, or suppressed by rapid host proliferation, respectively. In contrast to theoretical analyses used to assess models, we subjected model outputs to routine experimental data analyses and presentation to learn how to distinguish the models with empirical metrics. By doing so, we found that the models were remarkably similar. Virus and host parameters were similarly related to viral and host standing stocks. The relationship between lysis rates, host growth rates, viral and host abundances, and the virus: microbe ratio was conserved across all models. As a result, distinguishing between models appears to require experimental manipulation. Focusing on nutrient addition as a common experimental approach that also targets the mechanistic and mathematic differences between models, we found that simulating nutrient addition allowed us to bisect lytic and temperate models. In particular, lytic systems would experience no increase in host densities with nutrient addition but would show increasing instability of viral and host abundances. In contrast, temperate systems would remain consistently stable under nutrient addition but have increasing host densities. Building on this, we lay out a dichotomous key to allow researchers to determine whether lytic or temperate infection dynamics dominate their samples or ecosystem of interest using the commonly used approaches of counting viruses and hosts and measuring their responses to nutrient addition.

## 2. Methods

### 2.1. Models and Their Mechanistic Differences

Three mechanistically divergent theoretical models were compared (model parameters listed and summarized in Table 1). The models included a lytic Lotka–Volterra model with a logistic growth term (“Logistic Lotka-Volterra” [21]), a super-lytic model that enhances the viral killing of hosts when hosts are proliferating rapidly (“Weitz & Dushoff” [22]), and a sub-lytic/temperate model that suppresses the viral killing of hosts when hosts are proliferating rapidly (“Piggyback-the-Winner” [8]).

In all cases, the models comprise the following terms:

$\frac{dN}{dt}=$ host population growth—viral lysis—host viral-independent mortality

$\frac{dV}{dt}=$ viral production—viral decay

### 2.2. Logistic Lotka–Volterra Model

The Logistic Lotka–Volterra model is a basic predator–prey model [21]. This model has the following equations (see Table 1 for parameters):

(1) $$\frac{dN}{dt}=r\;N\;(1-\frac{N}{K})-\phi \;N\;V-m\;N$$

(2) $$\frac{dV}{dt}=\beta \;\phi \;N\;V-d\;V$$

These equations, like all the models, can be used to calculate steady-state solutions where the host and viral densities are unchanging (N* and V*, respectively; i.e., $\frac{dN}{dt}$ and $\frac{dV}{dt}\;$ = 0, assuming that N and V ≠ 0). These values are also known as equilibrium values because the host and viral densities are at equilibrium with each other and not generating oscillations. These densities presumably approximate the densities that would be sampled at any given time in the environment by direct counts of viruses and hosts, or serve as values that the host and viral densities might return to after perturbation [8,23,24,25]. When solving for steady-state solutions, these equations become the following:

(3) $$N^{*}=\frac{d}{\beta \;\phi }$$

(4) $$V^{*}=\frac{r}{\phi }-\frac{\;r\;N^{*}}{K\;\phi \;}-\frac{m}{\phi }$$

Therefore, in this model, host steady-state densities are driven by viral decay, burst size, and adsorption (i.e., steady-state host densities are an outcome of viral, not host parameters). Note that steady-state viral solutions (V*) in this and the following models are dependent on steady-state host densities (N*) and thus determined by these parameters as well. In addition, viral steady-state densities result from the host intrinsic growth rate, viral-independent mortality, and viral adsorption efficiency.

### 2.3. Weitz & Dushoff Model

In contrast with Piggyback-the-Winner, the “Weitz & Dushoff” model [22] enhances lysis and viral production when host densities are low and increasing. In this model, lysis and viral production are functions of host physiology. The Weitz & Dushoff model has the following equations (see Table 1 for parameters):

(5) $$\frac{dN}{dt}=r\;N\;(1-\frac{N}{K})-\phi \;N\;V\;(1-\frac{a\;N}{K})-m\;N$$

(6) $$\frac{dV}{dt}=\beta \;\phi \;N\;V\;(1-\frac{a\;N}{K})-d\;V\;$$

Note that this model introduces $1-\frac{a\;N}{K}$ elements to the lysis and viral production term, compared to Equation (2). The $1-\frac{N}{K}$ part of this element suppresses the lysis and production terms as $\frac{N}{K}\;\to \;1$. The *a* term determines how much lysis is suppressed as N approaches K and host growth slows.

When solved for steady-state solutions (i.e., $\frac{dN}{dt}$ = 0), Equations (9) and (10) become the following:

(7) $$N^{*}=\frac{\beta \;\phi \;K\;\pm \;\sqrt{{\left(\beta \;\phi \;K\right)}^{2}-(4\;\beta \;\phi \;a\;d\;K)}}{2\;\beta \;\phi \;a\;\;}$$

(8) $$V^{*}=\frac{r-m-r\;\frac{N^{*}}{K}}{\phi -\phi \;a\;\frac{N^{*}}{K}\;\;}$$

As a result of introducing the $1-\frac{a\;N}{K}$ element, there are many more parameters predicted to drive host and viral steady-state densities in this model, and the relationship between the parameters is much more complicated than in the other models. Further, the solution for N* involves a quadratic that therefore has two sets of solutions depending on whether the ± in Equation (7) is implemented as a plus or as a minus. In particular, implementing Equation (8) using a plus leads to the generation of pervasive negative steady-state values for viruses and hosts. Altogether, compared to other models, it is much less clear what drives viral and host steady-state densities and what those densities should be in this model.

### 2.4. Piggyback-the-Winner Model

The “Piggyback-the-Winner” model [8] simulates a temperate infection dynamic where viruses choose to not kill their rapidly growing hosts far below crowded and nutrient-limited densities. It does so by suppressing lysis at low host densities. The Piggyback-the-Winner model has the following equations (see Table 1 for parameters):

(9) $$\frac{dN}{dt}=r\;N\;(1-\frac{N}{K})-\phi \;N\;V-m\;N$$

(10) $$\frac{dV}{dt}=\beta \;\phi \;N\;V\;\frac{N}{K}-d\;V$$

Note that this model introduces $\frac{N}{K}$ to the viral production term, compared to Equation (2). This has the effect of suppressing viral production, and therefore infection, in low-density host conditions (i.e., $\frac{N}{K}<<1$). This delays the onset of lytic dynamics until hosts are dense (i.e., $\frac{N}{K}\;\to \;1$), presenting a de facto temperate to lytic transition as systems go from having low to high host densities.

When solved for steady-state solutions (i.e., $\frac{dN}{dt}$ = 0), Equations (5) and (6) become the following:

(11) $$N^{*}=\sqrt{\frac{K\;d}{\beta \;\phi }}$$

(12) $$V^{*}=\frac{r}{\phi }-\frac{\;r\;N^{*}}{K\;\phi \;}-\frac{m}{\phi }\;$$

Compared to the Logistic Lotka–Volterra model (Equations (3) and (4)), host steady-state densities in the Piggyback-the-Winner model are also driven by the ecosystem host carrying capacity (K), especially with N* rising with K. Viral steady-state solutions, V*, are the same as for Equation (4), but are driven differently by their dependence on N*, which varies between Equations (3) and (11), especially through the inclusion of K in the model.

### 2.5. The Effect of Varying All Parameters at Once

**Figure 1:**
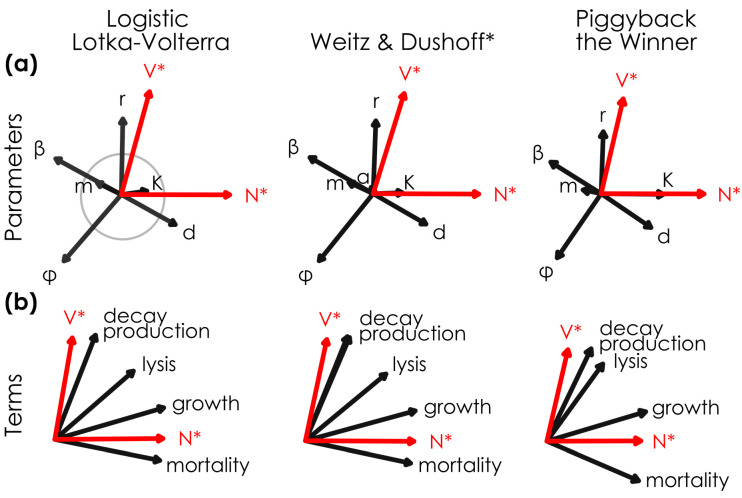
Mechanistically divergent lytic and sub-lytic models had similar relationships between viral density, host density, and permuted parameters and terms. The relationship between model (**a**) parameters, (**b**) terms, and steady-state viral and host abundances (V* and N*) in mechanistically-divergent models when parameters are randomly varied across 10,000 permutations (see Table 1 for the range of parameter values), where *n* = 7304, *n* = 7171, and *n* = 6275 solutions had positive virus and host densities in the Logistic Lotka–Volterra, Weitz & Dushoff models, and Piggyback-the-Winner models, respectively. Steady-state viral and host densities (red arrows) and parameters and terms (black arrows) are shown for lytic Logistic Lotka–Volterra, super-lytic Weitz & Dushoff, and sub-lytic Piggyback-the-Winner models. Vectors (i.e., arrows) were normalized across models; the gray circle in the top left panel has a loading value = 25 a.u. diameter. PCA outputs were rotated so that N* vectors had similar directions across models. * Note that the Weitz & Dushoff model results are for the ‘negative sign’ quadratic solution, as the ‘positive sign’ solution showed pervasive negative host and viral densities.

**Figure 2:**
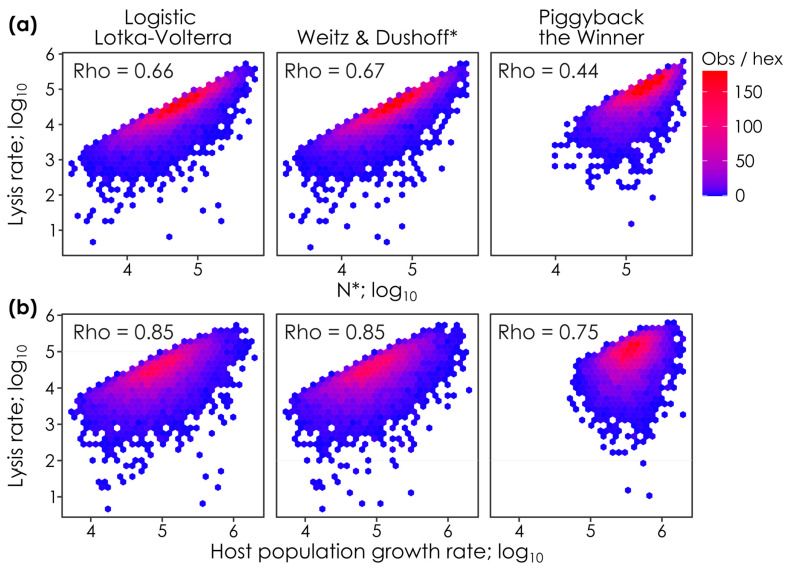
Lysis rates covary with steady-state host densities (N*) and host population growth rates in all models. The relationship between lysis rates and (**a**) steady-state host abundances (N*) and (**b**) host population growth rates when parameter values are permuted in lytic Logistic Lotka–Volterra, super-lytic Weitz & Dushoff, and sub-lytic Piggyback-the-Winner models. Each panel shows the outcomes of 10,000 parameter permutations (see Table 1), where *n* = 7304, *n* = 7171, and *n* = 6275 solutions had positive virus and host densities in the Logistic Lotka–Volterra, Weitz & Dushoff, and Piggyback-the-Winner models, respectively. Colors show the number of observations in each hex (i.e., observation density). Spearman’s Rho values, the monotonicity of the relationship between lysis rates and N* or host population growth rates, are shown on each panel. * Note that the Weitz & Dushoff model results are for the ‘negative sign’ quadratic solution, as the ‘positive sign’ solution showed pervasive negative host and viral densities. Note that correlations with Spearman’s Rho values < 0.2 are considered weak, 0.2. to 0.5 are moderate, and >0.5 strong [26].

**Figure 3:**
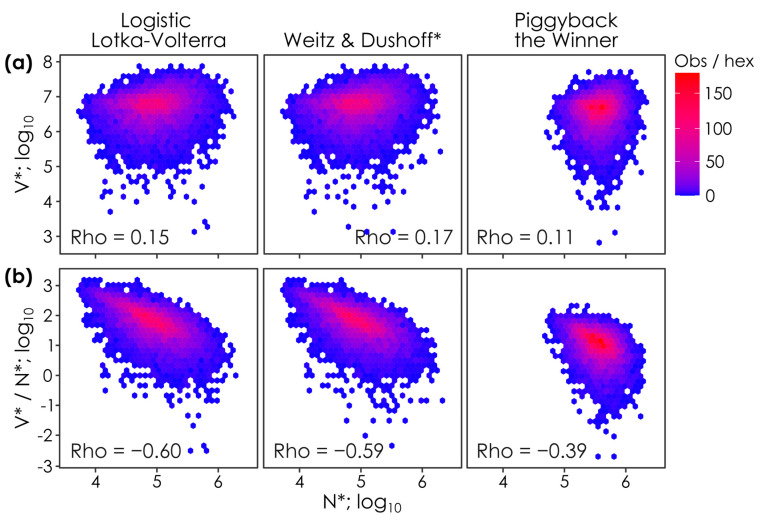
Host densities (N*) are positively correlated with viral densities (V*) and negatively correlated with virus-to-microbe ratios (V*/N*) in all models. The relationship between steady-state host (N*) and (**a**) viral densities (V*) and (**b**) virus-to-microbe ratios (V*/N*) when parameter values are permuted in lytic Logistic Lotka–Volterra, super-lytic Weitz & Dushoff, and sub-lytic/temperate Piggyback-the-Winner models. Each panel shows the outcomes of 10,000 parameter permutations (see Table 1), where *n* = 7304, *n* = 7171, and *n* = 6275 solutions had positive virus and host densities in the Logistic Lotka–Volterra, Weitz & Dushoff, and Piggyback-the-Winner models, respectively. Colors show the number of observations in each hex (i.e., observation density). Spearman’s Rho values, the monotonicity of the relationship between N* and V* or V*/N*, are shown on each panel. * Note that the Weitz & Dushoff model results are for the ‘negative sign’ quadratic solution, as the ‘positive sign’ solution showed pervasive negative host and viral densities. Note that correlations with Spearman’s Rho values < 0.2 are considered weak, 0.2. to 0.5 are moderate, and >0.5 strong [26].

To investigate how N* and V* values would look across different conditions (e.g., samples with different growth rates, burst sizes, etc.), we made arrays of random values for each parameter (r,  K, ϕ,  β,  d,  a) with 10-fold ranges, and solved for steady-state host (N*) and viral (V*) densities (see Table 1), building off Weitz et al. (2017) [20]. In total, we solved for 10,000 N* and V* values per model. We first quantified the effect of each parameter and term on N* and V* using Principal Component Analysis on the 10,000-solution dataset (PCA; prcomp() function with scale = TRUE; Figure 1). We then used Spearman’s Rho (the corr.test() function in R; with data log_10_-transformed and exact = FALSE) to summarize the relationship between N* and the host proliferation rate and lysis rates for each model across all 10,000 solutions (Figure 2), as well as for the relationship between N* and V* and the virus-to-microbe ratio (VMR; V*/N*; Figure 3). Note that the Spearman’s Rho coefficient quantifies how monotonic or consistent the correlation between two variables is and varies between −1 and 1 for strong negative and positive correlations, with 0 showing a lack of monotonic correlation. We considered correlations with different signs to be diagnostic between models (e.g., if the lytic models showed positive correlations but the temperate model had a negative correlation).

### 2.6. Effect of Altered Carrying Capacity (K) on Steady-State N* and V* and Stability

**Figure 4:**
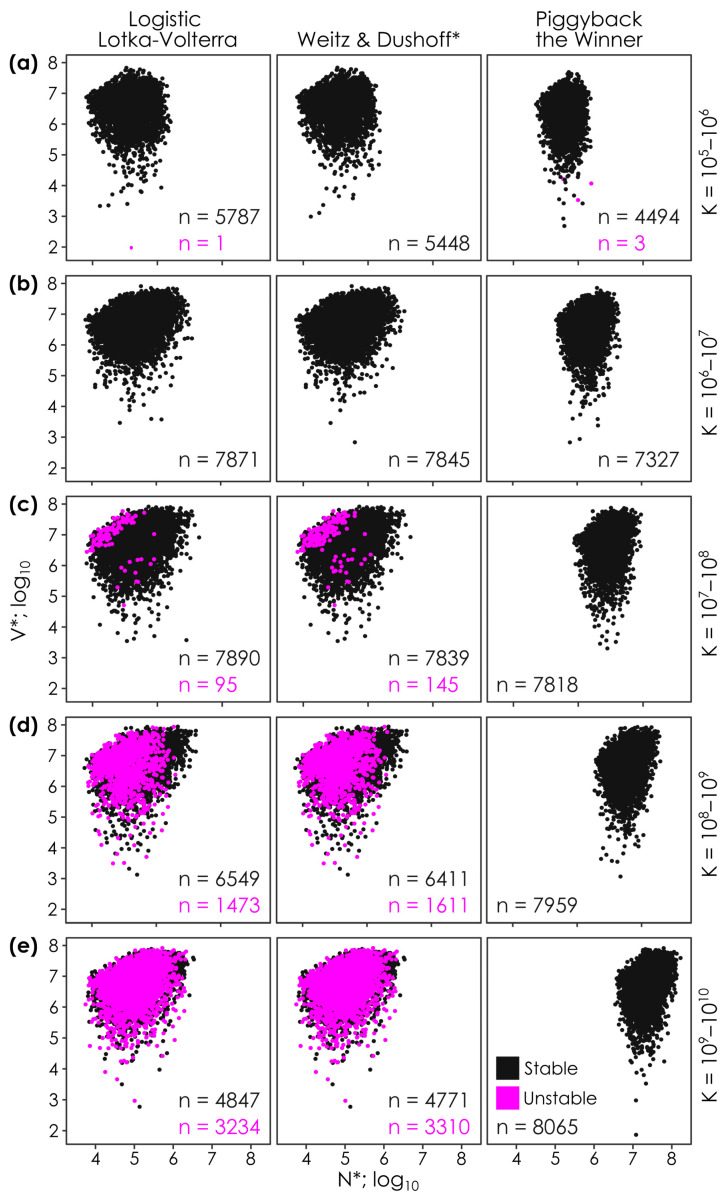
Increased nutrient availability (K) drives increasing instability in lytic models and increasing host densities in the temperate model. Increasing carrying capacity (equivalent to nutrient availability; K) from 1 × 10^5^ to 1 × 10^10^ cells per ml (**a**–**e**) leads to elevated host densities (N*) in the temperate Piggyback-the-Winner model, but not in the lytic Logistic Lotka–Volterra and Weitz & Dushoff models. In contrast, the simulated nutrient addition leads to increased instability in the lytic models (pink dots and text for unstable steady-states vs. black dots and text for stable steady states), with no change in stability in the temperate model. See each panel for the number of stable and unstable steady states (*n* = ; pink for unstable and black for stable solutions). Each panel shows the outcomes of 10,000 parameter permutations (see Table 1), where solutions had positive virus and host densities in each model. Note that K in this simulation assumes that the carrying capacity (i.e., nutrient availability) is constant or constantly added, in contrast with batch or pulse experiments. * Note that the Weitz & Dushoff model results are for the ‘negative sign’ quadratic solution, as the ‘positive sign’ solution showed pervasive negative host and viral densities.

To simulate the impact of nutrient enrichment, a widely used experimental approach that targets the mechanistic differences in the models, we modified the range of carrying capacity (K) for all models (Figure 4). Following the same methodology as in Section 2.5, we systematically increased K across multiple scenarios, allowing K to range between 1 × 10^5^–1 × 10^6^, 1 × 10^6^–1 × 10^7^, 1 × 10^7^–1 × 10^8^, 1 × 10^8^–1 × 10^9^, and 1 × 10^9^–1 × 10^10^ in each simulation. The ranges of all other parameters remained the same as in Section 2.5. We then computed the resulting steady-state host (N*) and viral (V*) densities to evaluate how increasing resource availability influences microbial and viral population dynamics under the same model framework. We performed stability analysis on these steady states to assess their robustness under different nutrient conditions. For each model, we computed the Jacobian matrix at the steady-state points and determined its eigenvalues. Calculations were conducted in Python using the sympy, numpy, and pandas package (Code is available at https://tinyurl.com/EmpiricistGuide). Each stability analysis resulted in two eigenvalues due to models being two dimensional systems. The stability of each steady-state point was classified as either stable or unstable based on the signs of the real parts of the eigenvalues (Figure 4; Supplementary Table S1). We categorized stable nodes and stable spirals as stable, and saddle points, unstable nodes, and unstable spirals as unstable. We did not observe any centers in our analysis, as no real parts of the eigenvalues were equal to 0.

### 2.7. Code and Implementation

All models were implemented in Python 3.6. Code is available at https://tinyurl.com/EmpiricistGuide. Steady-state predictions were generated for 10,000 iterations using Equations (3), (4), (7), (8), (11), and (12) with randomly generated parameter values within the ranges shown in Table 1 using the random.random() command for values < 1 and >0 and random.uniform() command for values > 1. Parameter values as well as predicted steady-state host and viral densities (N* and V*, respectively) and solutions for terms in Equations (1), (2), (5), (6), (9), and (10) were recorded. Terms (e.g., proliferation rate) were solved using the randomly generated parameters in each iteration. Stability analysis was conducted for each model by calculating Jacobians and eigenvalues using Python packages sympy, numpy, and pandas.

## 3. Results

### 3.1. Combined Effects of Parameter Variation

First, we were interested in how manipulating all parameters at once impacted viral and host steady-state densities (Figure 1). To address this, we ran 10,000 simulations for each model with randomized parameter values, solving for viral and host steady-state densities (V* and N*, respectively; see Table 1 for parameters; ‘shotgun’ analysis). This yielded *n* = 7304, *n* = 7171, and *n* = 6275 solutions with positive virus and host densities in the Logistic Lotka–Volterra, Weitz & Dushoff models, and Piggyback-the-Winner models, respectively. Note that only solutions from the ‘negative sign’ quadratic solution to the Weitz & Dushoff model were used, as the ‘positive sign’ outcomes were pervasively negative values for either host or virus densities.

We ran principal component analyses on the outputs from all models and the parameter values used (PCA; Figure 1). All models had surprisingly similar relationships between the parameters and steady-state viral and host densities (direction of vectors; Figure 1a). This means that the mechanistic differences between models are largely outweighed by their similarities. For example, the relationships between K and N* in the seemingly divergent Piggyback-the-Winner and Weitz & Dushoff models were similar. Further, the effect of the unique *a* term in the Weitz & Dushoff model had only minor effects (i.e., short arrow; Figure 1a).

Additionally, all models showed counter-intuitive patterns. For example, in all models we found that increasing the host growth rate *r* led to increased viral densities but had no effect on host densities (Figure 1a). Increasing viral burst size *β* led to the intuitive outcome of fewer hosts, but counterintuitively, the burst size had no relationship to viral densities (Figure 1a). Similarly, increasing the viral decay rate *d* led to increased hosts in all models, but, surprisingly, did not affect viral densities (Figure 1a). In all models, steady-state viral and host densities were orthogonal to each other, and therefore not directly related (Figure 1a).

### 3.2. Lysis Rates vs. Host Densities and Population Growth Rates Under Varying Host Parameters

The similarity between models also extended to term-level patterns (Figure 1b). Building on this, we investigated whether empirical researchers could differentiate the models by measuring and correlating host densities (N*) and host population growth rates with lysis rates (Figure 2a,b). By doing so, though, we observed that all models have similar relationships between these measures (i.e., all correlations had the same sign; Rho = 0.66, 0.67, 0.44 for N* vs. lysis rate and Rho = 0.85, 0.85, and 0.75 for proliferation vs. lysis rate for Logistic Lotka–Volterra, Weitz & Dushoff, and Piggyback-the-Winner models, respectively; all data log_10_-transformed), precluding their use to distinguish between lytic, super-lytic, and sub-lytic/temperate dynamics.

### 3.3. Steady-State Viral and Host Densities Under Varying Host Parameters

We then investigated whether the correlation of viral and host steady-state solutions resulting from the shotgun-varied parameters could be used to distinguish the models. As above, the models all had indistinguishable and positive relationships between N* and V* (Rho = 0.15, 0.17, and 0.11 for Logistic Lotka–Volterra, Weitz & Dushoff, and Piggyback-the-Winner models, respectively). Further, all models had negative correlations between VMR and host density (Figure 2b; Rho = −0.60, −0.59, and −0.39 in the Logistic Lotka–Volterra, Weitz & Dushoff, and Piggyback-the-Winner models, respectively).

### 3.4. Effect of Altered Carrying Capacity (K)

Given the common use of nutrient addition in empirical studies, and that the differences in the models center around the effect of distance to carrying capacity (e.g., the N*/K term), we investigated the effect of altering K on virus and host steady-state solutions and on their stability (Figure 4). By doing so, we found that N* did not appreciably rise (e.g., median N* = 7.51 × 10^4^ and 1.01 × 10^5^ cells per ml in the Logistic Lotka–Volterra model and median N* = 7.46 × 10^4^ and 1.01 × 10^5^ cells per ml in the Weitz & Dushoff model at K = 1 × 10^5^–1 × 10^6^, respectively) with nutrient addition in the lytic models, but rose ~ 100-fold in the temperate Piggyback-the-Winner model (e.g., median N* = 1.96 × 10^5^ and 2.21 × 10^7^ at K = 1 × 10^5^–1 × 10^6^ and 1 × 10^9^–1 × 10^10^, respectively; Figure 4). We also observed that instability increased in the lytic models with nutrient addition (e.g., *n* = 1:5787 and 0:5448 unstable–stable steady-states at K = 1 × 10^5^–1 × 10^6^ cells per ml rising to 3234:4847 and 3310:4771 unstable:stable steady-states at K = 1 × 10^9^–1 × 10^10^ cells per ml in the Logistic Lotka–Volterra and Weitz & Dushoff models, respectively; Figure 4). No such rise in instability was observed in the Piggyback-the-Winner model (3:4494 and 0:8065 unstable–stable steady states at K = 1 × 10^5^–1 × 10^6^ and 1 × 10^9^–1 × 10^10^, respectively).

## 4. Discussion

Viral infection of bacteria is one of the most common interactions on the planet. It is a major form of death in nature, with an estimated 10^23^ cells infected and lysed per second in the oceans [1]. Lytic infection can drive bacterial community composition by killing off lineages sensitive to infection and can drive bacterial evolution by selecting for resistant individuals [5,27,28]. This specific lysis of cells can also move large pools of resources between guilds. For example, the viral shunt describes the liberation of photosynthates and metabolites from algae to heterotrophic bacteria [29].

However, in addition to lytic infection, temperate infection can also take place. In temperate infection, the viruses decide whether to proceed with lysis after infecting the cell [30]. In this scenario, the viruses respond to host physiology in their decision-making and can reside for extended periods within the host genome. The lytic program can remain suspended until a physiological stress cue is received, inducing the restoration of the lytic program with intracellular viral production and subsequent lysis of the host. This suspended death can allow host densities to rise, in contrast to lytic infection which suppresses host densities [8].

Bacterial overgrowth has been associated with ecosystem degradation from coral reefs to the human lung and gut [15,31,32,33]. Given the divergent effects of lytic and temperate infection and how each lifestyle might be affecting ecosystem dysbiosis, understanding the role and prevalence of lytic and temperate infection has emerged as a central question in viral ecology. However, the fields of theoretical and empirical viral ecology—that lay out how these infection dynamics should look and that gather data on whether the dynamics are observed in nature, respectively—are disconnected and perhaps even incommensurate. This hinders theoreticians from refining improved models as well as empirical attempts to test the models. To address the gulf between theory and empirical approaches, we implemented three mechanistically divergent models—super-lytic, lytic, and sub-lytic/temperate—and explored their predictions as if they were empirical data to integrate theoretical and empirical approaches, hopefully accelerating progress in the field.

The models are surprisingly similar, especially given how mechanistically divergent they are. The similarity was observed across several analyses. First, we found that viral and host densities were linked similarly to model parameters like viral burst size, intrinsic growth rates, etc., and to model terms like the host population growth rate, viral production, etc. We also found that the relationship between host densities and viral densities and between host densities and the virus-to-microbe ratio were conserved across all models. Several of these, especially intrinsic and population growth rates and the virus-to-microbe ratio, have been used to differentiate between ecological dynamics like lytic and temperate infection and host infection/growth rate tradeoffs [20,34,35]. Our modeling suggests, however, that these metrics cannot effectively be used to differentiate even mechanistically divergent models.

**Figure 5:**
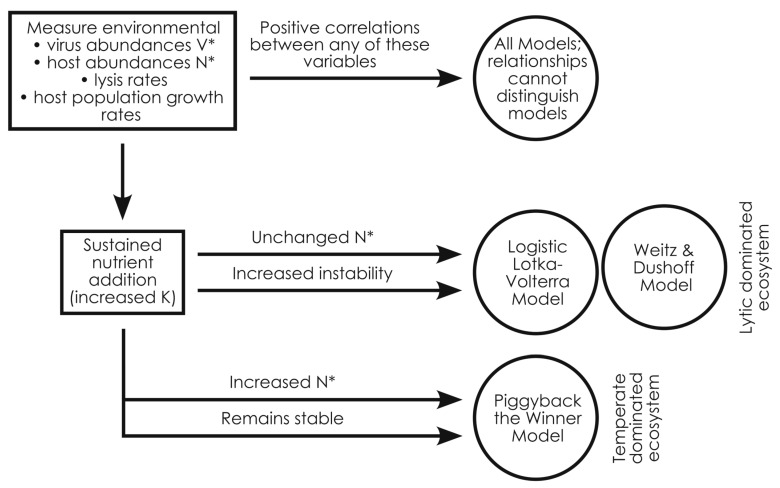
Dichotomous key for using empirically tractable methods to distinguish whether ecosystems are dominated by lytic or temperate dynamics. Correlations between many common empirical metrics are not able to distinguish between models (e.g., lysis rate goes up with N* and host population growth rate in all models, V*/N* declines with rising N* in all models, etc.). However, the dominance of lytic vs. temperate infection dynamics in a given ecosystem can be distinguished by the response to experimental nutrient addition, wherein lytic-dominated systems would see increased instability with no marked change in N* while temperate-dominated ecosystems would show elevated N* and sustained stability. Empirical approaches like quantifying viral and host densities in natural systems and nutrient addition are shown in boxes, arrows reflect the observed outcome of each approach, and the model indicated by the approach/outcomes is shown in circles.

However, the main mechanistic differences between the models hinge on how they drive lysis and viral production when cell densities are far from or close to carrying capacity (K). Nutrient addition—experimentally altering K to change the distance between the current cell abundance and carrying capacity—is also a commonly used empirical approach. We therefore focused on nutrient addition to try to distinguish the models. When we simulated nutrient addition, we were readily able to bisect the lytic and temperate models. The lytic models had no notable increase in host densities with nutrient addition, but became markedly less stable (Figure 5). In contrast, the temperate model showed elevated host densities and sustained stability with nutrient addition (Figure 5). It appears that, with all else being equal, temperate infection is required to allow ecosystems to have high host densities with nutrient addition (i.e., high carrying capacity).

Altogether our analyses sift through the remarkably similar predictions of the super-lytic Weitz & Dushoff, lytic Logistic Lotka–Volterra, or sub-lytic/temperate Piggyback-the-Winner models. Although it is likely that lytic and temperate infection takes place simultaneously in ecosystems, by highlighting the similarities and differences between the models, our work provides a guide for empiricists to assess whether their ecosystem or datasets are dominated by super-lytic, lytic, or sub-lytic infection dynamics through commonly used empirical metrics and experimental approaches (Figure 5). Ultimately, the ability to differentiate between these models using the tractable method of nutrient addition allows researchers to better describe the conditions favorable to lytic and temperate infections and to better predict the effects of viral infection in nature.

## Figures and Tables

**Table 1:** The symbols, values, units, and sources for parameters used in calculating steady-state solutions. Note that carrying capacity (K) was varied using the rand.int() Python command while all other parameters were varied using random.uniform(). Steady-state solutions were calculated for 10,000 permutations of randomly assigned parameter values in the ranges shown. Carrying capacity (K) ranges here refer to the analyses presented in Figure 1, Figure 2 and Figure 3, and varied from 1 × 10^5^ to 1 × 10^10^ cells per mL in the analysis shown in Figure 4. Data available at https://tinyurl.com/EmpiricistGuide (accessed on 21 March 2025).

Parameter	Symbol	Parameter Ranges	Units	Reference
Steady-state host density	N*		Cells per mL	
Steady-state viral density	V*		Viruses per mL	
Carrying capacity	K	3 × 10^5^ to 3 × 10^6^	Cells per mL	[8]
Intrinsic growth rate	*r*	10^−1^ to 10^0^	Cells per mL per time	[13]
Viral burst size	β	10^1^ to 10^2^	Viruses per infection	[13]
Viral adsorption coefficient	ϕ	10^−7^ to 10^−8^	Infections per encounter	[13]
Host natural mortality	*m*	5 × 10^−1^ to 5 × 10^−2^	Cells per mL	[13]
Viral decay	*d*	5 × 10^−1^ to 5 × 10^−2^	Viruses per mL	[13]
Suppression of lysis at K	*a*	5 × 10^−1^ to 5 × 10^−2^		

## Data Availability

Code and data are available at https://tinyurl.com/EmpiricistGuide.

## Supplementary Materials

The following supporting information can be downloaded at: https://www.mdpi.com/article/10.3390/v17040513/s1, Table S1: Stability categorization from eigenvalues.
