# Supplementary material for: Distinguishing Lytic and Temperate Infection Dynamics in the Environment
Source: Viruses. 2025 Apr 1;17(4):513. doi: 10.3390/v17040513 (PMC12031542; doi:10.3390/v17040513)
Supplement: Supplementary file 1 [file viruses-17-00513-s001.zip › viruses-3469399-supplementary.pdf]

Supplementary:

Table S1:

Eigenvalues	Stability Interpretation
Both real, negative	Stable
Both real, positive	Unstable
One positive, one negative	Saddle point – unstable equilibrium
Complex with real negative	Stable spiral – decaying oscillations
Complex with real positive	Unstable spiral – growing oscillations

Stability is categorized based on the signs of the real parts of the eigenvalues. If both real parts are negative, it is stable. Otherwise, it is unstable (since we categorize saddle points as unstable due to them being stable only in one direction).
